# Complete Genome Sequence of a Novel Human Gammapapillomavirus Isolated from a Cervical Swab in Luxembourg

**DOI:** 10.1128/genomeA.00114-18

**Published:** 2018-03-15

**Authors:** Ardashel Latsuzbaia, Marc Arbyn, Sankhadeep Dutta, Marc Fischer, Tarik Gheit, Jessica Tapp, Massimo Tommasino, Steven Weyers, Joël Mossong

**Affiliations:** a Epidemiology and Microbial Genomics, Laboratoire National de Santé, Dudelange, Luxembourg; b Unit of Cancer Epidemiology, Belgian Cancer Centre Scientific Institute of Public Health, Brussels, Belgium; c Infections and Cancer Biology Group, International Agency for Research on Cancer, Lyon, France; d Gynaecological Cytology, Laboratoire National de Santé, Dudelange, Luxembourg; e Department of Obstetrics and Gynecology, Ghent University Hospital, Ghent, Belgium

## Abstract

A novel human papillomavirus genotype was detected in a cervical swab specimen by next-generation sequencing after rolling circular amplification. It was fully cloned and characterized. The L1 open reading frame showed 77% nucleotide similarity with the closest genotype, HPV101, belonging to the gamma-6 species.

## GENOME ANNOUNCEMENT

Human papillomaviruses (HPVs) are small, circular, double-stranded DNA viruses that are linked to the malignant transformation of epithelia of various human sites (1). Currently, more than 200 HPV genotypes have been fully characterized, including five genera of papillomaviruses—alpha, beta, gamma, mu, and nu (2, 3). An HPV genome is considered a novel type if its L1 genomic region shares less than 90% similarity with the closest known HPV genotype (3, 4). Here, we describe the discovery of a novel gammapapillomavirus isolated from a cervical swab specimen (stored in PreservCyt liquid) taken from a 24-year-old woman participating in a vaccine efficacy study and vaccinated with two doses of quadrivalent HPV vaccine. The cytological result was negative for intraepithelial lesion and malignancy.

Following DNA extraction using the Qiagen DNA minikit (Qiagen, Germany), DNA was enriched using rolling circle amplification (RCA) according to the manufacturer’s instructions (illustra TempliPhi, GE Healthcare, USA) (5). A library was prepared using the Nextera XT kit (Illumina, Inc., USA), followed by next-generation sequencing on the Illumina MiniSeq platform (Illumina, Inc.). Of a total of 1,226,786 reads, 40,737 (3.3%) were mapped using BBMap within Geneious version 11 (6) to 286 known HPV genotypes downloaded from the Papillomavirus Episteme database (2), which was accessed on 24 November 2017. Reads mapped to 11 different genotypes (51, 58, 66, 67, 73, 87, 89, 90, 91, 101, and MF588697) using a minimal threshold of 10 reads. *De novo* assembly using SPAdes version 3.10.0 in Geneious yielded 6 completely known HPV genotypes (51, 66, 67, 87, 89, and MF588697) and 1 novel genotype (16031680A). Long-range PCR was performed with the RCA product using TaKaRa-LA *Taq* DNA polymerase (TaKaRa Bio, Inc.), which was cloned using the TOPO XL PCR cloning kit (Invitrogen, USA) and sequenced. The clone was submitted to the HPV reference center in Stockholm.

The L1 open reading frame (ORF) of HPV isolate 16031680A showed 77% nucleotide identity with the closest genotype, HPV101, belonging to the gamma-6 species, and analysis using BLAST showed 77% pairwise identity for the full genome. The complete genome of this novel HPV genotype consists of 7,313 nucleotides with a G+C content of 42.6%. It has four early (E1, E2, E4, and E7) and two late (L1 and L2) ORFs. The ORF coding the early protein E6 is absent, as it is for other known members of the gamma-6 species, namely, 101, 103, and 108 (7–9). The long control region of 735 nucleotides found between the L1 and E7 genes contains one TATA box (TATAAA) and three palindromic E2-binding sites (ACC-N_6_-GGT). One conserved zinc-binding domain was identified in the E7 protein [CxxC(x)_29_CxxC], and the ATP-binding site of the ATP-dependent helicase (GPPDTGKS) was identified in the carboxy-terminal region of E1 (7).

To conclude, we discovered a novel genotype of the gamma-6 papillomavirus species. Members of this species are adapted to the mucosal niche (9) and may be associated with low-grade (8) and high-grade cervical lesions (7). The clinical relevance and prevalence of gamma-6 genotypes deserves further investigation.

### Accession number(s).

The complete genome sequence for human gammapapillomavirus isolate 16031680A is available in GenBank under accession number MG813996.

## References

[1] IARC Working Group on the Evaluation of Carcinogenic Risks to Humans. 2012 Biological agents. Volume 100 B. A review of human carcinogens. IARC Monogr Eval Carcinog Risks Hum 100:1–441. PMC4781184 23189750

[2] Van Doorslaer K, Li Z, Xirasagar S, Maes P, Kaminsky D, Liou D, Sun Q, Kaur R, Huyen Y, McBride AA 2017 The Papillomavirus Episteme: a major update to the papillomavirus sequence database. Nucleic Acids Res 45:D499–D506. doi:10.1093/nar/gkw879. 28053164 PMC5210616

[3] Bernard HU, Burk RD, Chen Z, van Doorslaer K, Zur Hausen H, de Villiers EM 2010 Classification of papillomaviruses (PVs) based on 189 PV types and proposal of taxonomic amendments. Virology 401:70–79. doi:10.1016/j.virol.2010.02.002. 20206957 PMC3400342

[4] de Villiers EM, Fauquet C, Broker TR, Bernard HU, Zur Hausen H 2004 Classification of papillomaviruses. Virology 324:17–27. doi:10.1016/j.virol.2004.03.033. 15183049

[5] Rector A, Tachezy R, Van Ranst M 2004 A sequence-independent strategy for detection and cloning of circular DNA virus genomes by using multiply primed rolling-circle amplification. J Virol 78:4993–4998. doi:10.1128/JVI.78.10.4993-4998.2004. 15113879 PMC400362

[6] Kearse M, Moir R, Wilson A, Stones-Havas S, Cheung M, Sturrock S, Buxton S, Cooper A, Markowitz S, Duran C, Thierer T, Ashton B, Meintjes P, Drummond A 2012 Geneious Basic: an integrated and extendable desktop software platform for the organization and analysis of sequence data. Bioinformatics 28:1647–1649. doi:10.1093/bioinformatics/bts199. 22543367 PMC3371832

[7] Chen Z, Schiffman M, Herrero R, Desalle R, Burk RD 2007 Human papillomavirus (HPV) types 101 and 103 isolated from cervicovaginal cells lack an E6 open reading frame (ORF) and are related to gamma-papillomaviruses. Virology 360:447–453. doi:10.1016/j.virol.2006.10.022. 17125811 PMC1885239

[8] Nobre RJ, Herráez-Hernández E, Fei J-W, Langbein L, Kaden S, Gröne H-J, de Villiers E-M 2009 E7 oncoprotein of novel human papillomavirus type 108 lacking the E6 gene induces dysplasia in organotypic keratinocyte cultures. J Virol 83:2907–2916. doi:10.1128/JVI.02490-08. 19153227 PMC2655592

[9] Van Doorslaer K, McBride AA 2016 Molecular archeological evidence in support of the repeated loss of a papillomavirus gene. Sci Rep 6:33028. doi:10.1038/srep33028. 27604338 PMC5015084

